# Stratified analysis based on VAS scores to assess the effect of acupoint application therapy in children with chronic coughs of varying severity-a randomized controlled study

**DOI:** 10.3389/fmed.2025.1580229

**Published:** 2026-03-23

**Authors:** Danxia Ni, Liyun Zhong, Xinping Liu, Wenjing Lai, Jingyi Mai, Weiyi Dai, Meifeng Xie, Lifang Lei

**Affiliations:** 1 Department of Pediatrics, Guangzhou Twelfth People’s Hospital, Guangzhou, Guangdong, China; 2 Department of Rehabilitation Medicine, Guangzhou Twelfth People’s Hospital, Guangzhou, Guangdong, China; 3 Department of Pediatric Massage, Guangdong Provincial Hospital of Traditional Chinese Medicine, Guangzhou, Guangdong, China; 4 Department of Acupuncture, Guangdong Provincial Hospital of Traditional Chinese Medicine, Guangzhou, Guangdong, China

**Keywords:** acupoint application therapy, child, chronic cough, stratified analysis, visual analogue scale

## Abstract

**Methods:**

In this randomized controlled trial, one hundred children with chronic cough were allocated to either a control group (*n* = 50) receiving conventional bronchodilator and inhaled corticosteroid therapy, or an observation group (*n* = 50) receiving conventional therapy plus acupoint application. Outcomes including cough symptom scores, Leicester Cough Questionnaire (LCQ), and visual analogue scale (VAS) for pain were assessed at baseline and after a 2-week intervention. The primary analysis employed multivariate regression to determine the independent effect of the intervention, adjusting for baseline scores and allergy history.

**Background:**

This study explores the efficacy of acupoint application therapy in children with chronic coughs of varying severity.

**Results:**

After treatment, both groups showed significant within-group improvements (all *p* < 0.001). However, inter-group comparisons revealed that the observation group achieved superior outcomes, with significantly lower post-treatment daytime cough scores (2.46 ± 0.28 vs. 2.68 ± 0.24, *p* < 0.001), nighttime cough scores (2.14 ± 0.36 vs. 3.04 ± 0.54, *p* < 0.001), and VAS scores (3.89 ± 1.35 vs. 5.52 ± 1.87, *p* < 0.001), alongside a higher LCQ score (15.61 ± 3.22 vs. 14.45 ± 3.17, *p* = 0.034). Multivariate analysis confirmed acupoint application as an independent factor associated with these improvements (adjusted mean difference for daytime cough: -0.21, *p* < 0.001) and a higher treatment response (adjusted odds ratio: 4.72, *p* = 0.007). Furthermore, within the observation group, patients with severe baseline pain exhibited a greater magnitude of improvement across all scores compared to those with mild pain (ΔVAS: 3.39 vs. 1.78, *p* = 0.017). The overall response rate was 85% in the observation group versus 81% in the control group (*p* = 0.041). No adverse reactions were reported.

**Conclusion:**

Acupoint application therapy is clinically effective in children with chronic coughs, with more pronounced efficacy observed in patients experiencing severe pain.

## Background

Coughing can occur spontaneously or voluntarily and is considered chronic when it persists for over 4 weeks in children or more than 8 weeks in adults (1). Chronic cough affects approximately 5–10% of adult population (2). A meta-analysis estimated the global prevalence of chronic cough in the general adult population as about 10% (2). It was more prevalent in Europe, America and Oceania than in Asia and Africa. It may be post-infectious and/or caused by asthma, persistent bacterial bronchitis and gastro-oesophageal reflux disease (3). In particular, coughing can substantially impact a child’s quality of life (QoL), leading to sleep disorders, absenteeism and stress for both the child and their caregivers (4). Conventional treatments (drug treatment) focus on addressing the underlying causes of cough; however, the effect is limited in more than 70% of patients with chronic cough (5). Therefore, an effective alternative treatment approach is required, especially in cases where the cough is resistant to conventional treatments. In such situations, traditional Chinese medicine (TCM) treatment, including herbal medicine and/or acupuncture, has long been used for cough (6, 7). Compared with traditional herbal medicine and acupuncture, children exhibit better acceptance of and compliance with external treatment methods such as acupoint application (8). Acupoint application has demonstrated evident efficacy in children with chronic cough (9), substantially improving chronic cough symptoms and patient satisfaction (10). Coughing is often accompanied by pain (11), and the severity of cough symptoms in children is frequently assessed using the face, legs, activity, cry, consolability behavioral tool (12).

It is hypothesised that the efficacy of acupoint application in chronic cough may vary according to pain severity. Therefore, this study was designed to explore the effects of acupoint application on chronic cough of differing pain severity, based on validation of its efficacy in chronic cough, thereby providing guidance for the clinical application of this therapy.

## Materials and methods

### General data

**Figure 1:**
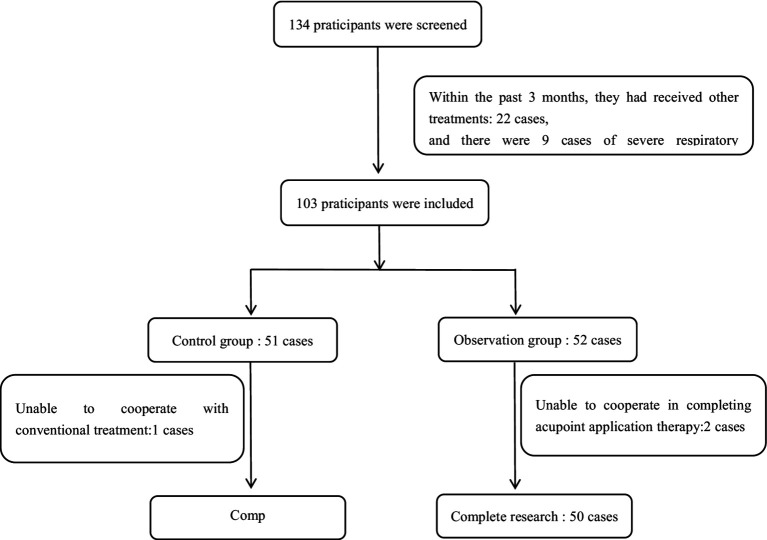
The screening and exit of participants.

This study conformed to the CONSORT guidelines. One hundred children with chronic coughs from the Paediatrics Department of the Twelfth People’s Hospital of Guangzhou, between June 2023 and October 2024, were selected as participants for this study. They were randomly divided into a control group (*n* = 50) and an observation group (*n* = 50) using a random number table. In previous work, the research team found that children under 4 years old have a high incidence of adverse reactions after using acupoint application therapy. Students after the age of 12 have difficulty adhering to treatment due to their busy studies and poor compliance. Therefore, this study included children aged 4–12 years old. Retrieve random numbers from a random number table. Statisticians who do not participate in patient screening will pack the random sequence into sealed, numbered opaque envelopes. When the patient meets the inclusion criteria, the researcher will open the corresponding numbered envelopes in the order of enrollment to obtain the grouping results. Specifically, the control group received conventional treatment, and the observation group received acupoint application therapy in addition to the conventional treatment. All participants underwent 2 weeks of intervention. Normally, the sample size should be 5 to 10 times the number of items in the survey questionnaire (13). The number of items on the Leicester Cough Questionnaire (LCQ) used in this study was 19; therefore, the appropriate sample size range was 95–190. This study included a total of 100 participants. The screening process for participants is shown in Figure 1. This study was conformed by the Twelfth People’s Hospital of Guangzhou (Approval number: 2023082), and all participants have signed informed consent forms. This study was conducted by registering on the ISRCTN website^1^ with registration number: ISRCTN83971033.

### Diagnostic criteria

The following diagnostic criteria were used to determine participant eligibility:

Traditional chinese medicine diagnostic criteria (14): referencing the diagnosis of cough (the syndrome of qi deficiency of the lung and spleen) outlined in the national textbook *Pediatrics in Chinese Medicine*, edited by Wang Shouchuan, the criteria are as follows: (1) main symptoms – cough, primarily irritative dry cough with white, sticky phlegm; (2) secondary symptoms – throat itchiness, throat dryness, pale complexion, fatigue, spontaneous sweating, aversion to cold, poor appetite and easy susceptibility to infections; (3) tongue appearance – pale and tender tongue with tooth marks on the edges and (4) pulse condition – thin and weak pulse with light red fingerprints. It should be noted that the main symptom must be present, accompanied by two or more secondary symptoms, and combined with tongue and pulse signs to diagnose the syndrome of qi deficiency of the lung and spleen.
Western medicine diagnostic criteria (15): referencing the *Clinical Practice Guidelines for Diagnosis and Treatment of Cough in Children (2021 Edition)*, the criteria are as follows: (1) medical history – a definite history of recent respiratory tract infection; (2) physical examination – cough lasting over 4 weeks, presenting as irritative dry cough or accompanied by a small amount of white, sticky phlegm; (3) chest X-ray – no obvious abnormalities or only increased lung markings; (4) lung function tests – recommended for children over 6 years of age; (5) further evaluation – other diagnoses should be considered if the cough persists beyond 8 weeks; (6) cause – chronic cough due to other aetiologies.

The inclusion criteria included the following: (1) patients aged 4–12 years; (2) patients meeting the relevant diagnostic criteria of both TCM and Western medicine as stated above; (3) patients without obvious organic diseases or other severe complications and (4) patients and their parents or guardians willing to participate in the study and able to provide informed consent.

The exclusion criteria included the following: (1) patients with severe respiratory complications, e.g., pneumonia or bronchial asthma; (2) patients who had received other specific treatments for cough, e.g., antibiotic therapy or immunomodulatory therapy, within the past 3 months and (3) patients unable to cooperate with acupoint application procedures due to age, cognitive ability or other reasons.

### Intervention methods

In the past treatment process, researchers have found that patients with chronic cough often experience chronic inflammation and feel sore throat when coughing. Therefore, this study focused on the condition of sore throat during coughing. Before receiving treatment, all participants underwent evaluation of cough symptoms the degree of throat pain when coughing occurs, which were used as baseline data to assess efficacy.

#### Control group

After 2 weeks of diagnostic bronchodilator therapy and empirical inhaled corticosteroids (ICS), a reassessment was performed. The following bronchodilators were used: ipratropium bromide solution for inhalation (Joincare Pharmaceutical Group Industry Co. Ltd., approval no.: GYZZ H20203454, product specification: 0.5 mg/vial), terbutaline sulphate solution for nebulisation (Joincare Pharmaceutical Group Industry Co. Ltd., approval no.: GYZZ H20223371, product specification: 5 mg/vial) and salbutamol sulphate solution for inhalation (Suzhou Homesun Pharmaceutical Co. Ltd., approval no.: GYZZ H20203292, product specification: 5 mg/vial). Empirical ICS included: budesonide suspension for inhalation (Sichuan Purity Pharmaceutical Co. Ltd., approval no.: GYZZ H20213286, product specification: 1 mg/vial) and fluticasone propionate (GlaxoSmithKline Pty Ltd., Imported Drug registration no.: H20170361, product specification: 0.5 mg/vial). Due to differences in etiology and symptoms among different participants, the control group participants did not receive a unified treatment plan. Treatment involved a single medication or a combination of two to three medications administered via nebulisation, with dosages and methods adjusted according to clinical needs, along with routine symptomatic treatments for allergies, spasms and asthma.

#### Observation group

In addition to the treatment given to the control group, the observation group underwent acupoint application therapy. Referencing the external treatment methods for cough (the syndrome of qi deficiency of the lung and spleen) outlined in the national textbook *Pediatrics in Chinese Medicine*, edited by Wang Shouchuan, the acupoint application was performed as follows: 12 g of cinnamon, 16 g of clove, 10 g of cassia twig, 15 g of frankincense, 10 g of *Codonopsis pilosula*, 10 g of *Astragalus membranaceus* and 30 g each of *Angelica sinensis*, safflower, red peony, *chuanxiong* and tuberculate *Speranskia* herb were ground into fine powder (100 mesh) and sealed in a bottle for later use. When used, the powder was mixed with honey to form a paste or shaped into pills the size of peanuts, followed by placing the prepared medicine in the centre of a sterile dressing.

#### Acupoint selection

Days 1–2: Pills were used at the acupoints of Tiantu-CV22 and Danzhong-CV17; pastes were used bilaterally at both Dingchuan-EXB1 and Feishu-BL13. Days 3–5: Pastes were used at the acupoints of Zhongwan-CV12, Shenque-CV8, Qihai- CV6 and Guanyuan-CV4.

#### Selection of application method

The skin was cleaned with alcohol, and the patient lay supine to ensure stable application of the medicine. The sterile dressing was then placed on the designated acupoints and gently pressed to ensure full contact between the medicine and the acupoints, followed by securing it with adhesive tape. The application was performed once daily and removed after approximately 4 h, with a treatment course of 5–7 days. The total intervention lasted 2 weeks.

#### Precautions

(1) Raw and cold food, seafood and spicy foods were prohibited during the application period; (2) The duration of application should be based on the individual’s tolerance, with each application maintained for 4–6 h. The application should be discontinued immediately if skin allergies, intolerable itching or pain occur; (3) After applying the medicine, the application area should be kept dry; (4) A bath with warm water could be taken 6 h after application.

### Efficacy evaluation

In order to avoid the influence of the subjective consciousness of evaluators on the results, two full-time personnel were arranged to conduct efficacy evaluations. The personnel conducting the efficacy evaluation did not participate in other aspects of this study, such as enrolment and intervention.

#### Clinical efficacy

The evaluation was performed according to the *Consensus Opinion of Traditional Chinese Medicine Diagnosis and Treatment Experts on Cough* (16). Symptoms such as cough, expectoration, wheezing and susceptibility were considered cured if they disappeared and dry or moist rales were no longer heard upon auscultation = *cured*; if the main symptoms were substantially alleviated, there was no onset in winter or the onset was mild or the symptoms during onset were easier to control than before, with clear breath sounds and reduced phlegm = *substantially effective*; if the frequency of onset decreased and symptoms improved = *improved*; if there was no substantial change or aggravation of symptoms before and after treatment = *ineffective*. The overall response rate = (cured + substantially effective + improved) / total cases × 100%.

#### Improvement of cough symptoms

Cough symptoms were evaluated using a cough symptom scoring system and the LCQ (17) at baseline and 2 weeks post-intervention. Specifically, the scoring system comprises daytime and night-time assessments. Daytime scores are defined as follows: 0 – no cough; 1 – one to two brief coughs; 2 – more than two brief coughs; 3 – frequent coughs that do not affect daily life; 4 – frequent coughs that affect daily life and 5 – severe cough that makes daily life difficult. The night-time score includes: no cough, cough only when awake or about to sleep, waking once or early due to cough, frequent night-time awakenings due to cough, coughing most of the night and severe cough making it difficult to sleep, also scored from 0 to 5 points. The LCQ scale consists of 19 items, each scored from 1 to 7 points, covering physical, psychological and social aspects, with a total score of 1–21 points. Lower scores indicate a greater impact of cough on the QoL of affected children.

#### Visual analogue scale (VAS)

The VAS score was used to assess pain in both groups before and after treatment, with the score positively correlated with pain severity. The score ranges from 0 to 10 points, with 0 points indicating no pain, ≤3 points indicating mild pain, 4–6 points indicating moderate pain and ≥7 points indicating severe pain (18).

#### Adverse reactions

The occurrence of adverse reactions during treatment was observed, including skin allergies, nausea, vomiting and diarrhoea. Adverse reactions due to acupoint application during the study were recorded in detail, along with a comparison of incidence rates. The incidence rate of adverse reactions = number of adverse reactions in children/total cases × 100%.

### Statistical methods

Statistical analysis was performed using SPSS software (version 23.0; IBM, Armonk, NY, United States). The Shapiro–Wilk test was used to assess the normality of continuous data, and Bartlett’s test was used to assess the homogeneity of variances. Normally distributed measurement data were expressed as the mean ± standard deviation and analyzed using the t-test (paired t-test for within-group comparisons and independent t-test for between-group comparisons). Non-normally distributed data were expressed as quartiles and analyzed using non-parametric tests (Mann–Whitney U test for between-group comparisons). Categorical data were expressed as numbers (percentages) and compared using the χ^2^ test or Fisher’s exact test, as appropriate.

To further validate the primary findings and control for potential confounding factors, multivariate analyses were conducted. Multiple linear regression models were employed for continuous outcomes (post-treatment daytime cough score, nighttime cough score, QoL score, and VAS score), and a multiple logistic regression model was employed for the binary outcome (treatment response). All models included the following covariates: treatment group (observation vs. control), the baseline value of the corresponding outcome variable, and history of allergy (which showed a significant baseline imbalance). The results of the regression analyses are presented as adjusted mean differences (aMD) with 95% confidence intervals (CI) for linear regression, and adjusted odds ratios (aOR) with 95% CI for logistic regression. A two-sided *p* value of < 0.05 was considered statistically significant for all tests.

## Results

### General data

Baseline characteristics of the participants are summarized in Table 1. There were no significant differences between the observation and control groups in gender distribution (male/female: 22/28 vs. 27/23, χ^2^ = 1.76, *p* = 0.185), age (8.60 ± 2.49 years vs. 8.86 ± 2.61 years, t = −0.52, df = 98, *p* = 0.108), or course of disease (3.80 ± 1.95 days vs. 4.62 ± 6.61 days, U = 1150.5, *p* = 0.241). However, a significant difference was observed in the history of allergy (8 vs. 0, Fisher’s exact test, *p* < 0.001). For the etiology of chronic cough, no significant differences were found between groups in post-infectious cough (28 vs. 31, χ^2^ = 0.96, *p* = 0.328), asthma (6 vs. 4, Fisher’s exact test, *p* = 0.149), persistent bacterial bronchitis (4 vs. 6, Fisher’s exact test, *p* = 0.151), gastro-oesophageal reflux disease (1 vs. 3, Fisher’s exact test, *p* = 0.228), allergic cough (7 vs. 3, Fisher’s exact test, *p* = 0.084), or other causes (4 vs. 3, Fisher’s exact test, *p* = 0.219). All participants completed the study, with no withdrawals reported.

**Table 1:** General data of participants.

Indicator	Observation group (*n* = 50)	Control group (*n* = 50)	Statistic	*p* value
Gender, M/F (*n*)	22/28	27/23	χ^2^ = 1.76	0.185
Age (years)	8.60 ± 2.49	8.86 ± 2.61	t = −0.52*	0.108
Course of disease (days)	3.80 ± 1.95	4.62 ± 6.61	U = 1150.5†	0.241
History of allergy, *n*	8	0	Fisher’s exact test	<0.001
Etiology of chronic cough, *n*				
Post-infectious cough	28	31	χ^2^ = 0.96	0.328
Asthma	6	4	Fisher’s exact test	0.149
Persistent bacterial bronchitis	4	6	Fisher’s exact test	0.151
Gastro-oesophageal reflux disease	1	3	Fisher’s exact test	0.228
Allergic cough	7	3	Fisher’s exact test	0.084
Others	4	3	Fisher’s exact test	0.219

Data are presented as mean ± standard deviation or number (*n*). M/F: male/female. *t-value from independent t-test, df = 98. †U-value from Mann–Whitney U test.

### Changes in symptoms, QoL, and pain before and after treatment

Baseline characteristics, including daytime cough score, nighttime cough score, QoL, and VAS score, were comparable between the control and observation groups (all *p* > 0.05; Table 2).

**Table 2:** Changes in symptoms, QoL, and pain pre- and post-treatment in the control and observation groups.

Indicator	Observation time	Control group (*n* = 50)	Observation group (*n* = 50)	Between-group statistic (post-treatment)	*p* value (between-group, post-treatment)
Daytime cough score	Pre-treatment	4.32 ± 0.64	4.65 ± 0.58	t = −0.78†	0.219
	Post-treatment	2.68 ± 0.24*	2.46 ± 0.28*	t = 4.37†	<0.001
Nighttime cough score	Pre-treatment	4.42 ± 0.71	4.51 ± 0.24	t = −0.86†	0.196
	Post-treatment	3.04 ± 0.54*	2.14 ± 0.36*	t = 9.83†	<0.001
Quality of life (QoL)	Pre-treatment	8.76 ± 1.93	8.72 ± 2.31	t = 0.09†	0.464
	Post-treatment	14.45 ± 3.17*	15.61 ± 3.22*	t = −1.84†	0.034
VAS score	Pre-treatment	6.83 ± 2.71	6.76 ± 2.23	U = 1220.0‡	0.441
	Post-treatment	5.52 ± 1.87*	3.89 ± 1.35*	U = 720.5‡	<0.001
Response rate	Post-treatment	81% (40/50)	85% (42/50)	χ^2^ = 4.17§	0.041

Data are presented as mean ± standard deviation or percentage (n/N). *Indicates a statistically significant difference from the pre-treatment score within the same group (*p* < 0.001, paired t-test for all within-group comparisons). † Independent t-test. ‡ Mann–Whitney U test. § Chi-square test.

Following the 2-week intervention, both groups demonstrated significant improvements in all outcome measures. Specifically, the control group showed reductions in daytime cough score (from 4.32 ± 0.64 to 2.68 ± 0.24; t = 17.82, *p* < 0.001) and nighttime cough score (from 4.42 ± 0.71 to 3.04 ± 0.54; t = 12.15, *p* < 0.001). Similarly, the observation group exhibited significant decreases in daytime cough score (from 4.65 ± 0.58 to 2.46 ± 0.28; t = 26.41, *p* < 0.001) and nighttime cough score (from 4.51 ± 0.24 to 2.14 ± 0.36; t = 43.17, *p* < 0.001). Inter-group comparisons after treatment revealed that the observation group achieved significantly lower scores than the control group in both daytime (t = 4.37, *p* < 0.001) and nighttime cough (t = 9.83, *p* < 0.001).

Regarding QoL and pain, both groups improved significantly from baseline (all within-group *p* < 0.001). The QoL score increased to 14.45 ± 3.17 in the control group and to 15.61 ± 3.22 in the observation group, while the VAS score decreased to 5.52 ± 1.87 and 3.89 ± 1.35, respectively. Post-treatment, the observation group had a significantly higher QoL score (t = −1.84, *p* = 0.034) and a significantly lower VAS score (U = 720.5, *p* < 0.001) compared to the control group.

Furthermore, the overall response rate in the observation group (85%, 42/50) was significantly higher than that in the control group (81%, 40/50; χ^2^ = 4.17, *p* = 0.041).

### Changes in symptoms, QoL, and pain in patients with different pain severity in the observation group

Within the observation group, patients across all pain severity strata (mild, moderate, and severe) demonstrated significant improvements in daytime cough, nighttime cough, QoL, and VAS scores after treatment compared to their baseline values (all within-group *p* < 0.001, paired t-tests, Table 3).

**Table 3:** Changes in symptoms, QoL, and pain stratified by baseline pain severity in the observation group.

Indicator	Observation time	Mild (*n* = 15)	Moderate (*n* = 21)	Severe (*n* = 14)	Test for group effect on *Δ* values	*p* value
Daytime cough score	Pre-treatment	3.92 ± 0.83	4.65 ± 0.58	5.16 ± 0.75		
	Post-treatment	2.53 ± 0.34*	2.46 ± 0.28*	2.24 ± 0.48*		
	Change (Δ)	1.39 ± 0.83	2.19 ± 0.57	2.92 ± 0.81*a	*F* = 4.81†	0.013
Nighttime cough score	Pre-treatment	4.23 ± 0.54	4.51 ± 0.24	4.91 ± 0.64		
	Post-treatment	2.19 ± 0.32*	2.14 ± 0.36*	1.81 ± 0.72*		
	Change (Δ)	2.04 ± 0.58	2.37 ± 0.44	3.10 ± 1.04*a	*F* = 6.74†	0.003
Quality of life (QoL)	Pre-treatment	8.91 ± 3.01	8.72 ± 2.31	8.21 ± 2.63		
	Post-treatment	14.24 ± 2.52*	15.61 ± 3.22*	16.01 ± 3.42*		
	Change (Δ)	5.33 ± 2.86	6.89 ± 3.45	7.80 ± 3.85*a	*F* = 5.12†	0.010
VAS score	Pre-treatment	6.05 ± 2.67	6.76 ± 2.23	7.02 ± 2.73		
	Post-treatment	4.27 ± 2.24*	3.89 ± 1.35*	3.63 ± 1.45*		
	Change (Δ)	1.78 ± 2.15	2.87 ± 2.31	3.39 ± 2.58*a	*F* = 5.89†	0.005
Total response rate	Post-treatment	85% (13/15)	82% (17/21)	84% (12/14)	χ^2^ = 0.08‡	0.961

Data are presented as mean ± standard deviation or percentage (n/N). Δ = Pre-treatment score − Post-treatment score. * Indicates a statistically significant difference from the pre-treatment score within the same subgroup (*p* < 0.001, paired t-test). a Indicates that the change (Δ) is significantly greater than that in the mild pain subgroup (*p* < 0.05, post-hoc Tukey test). † One-way ANOVA. ‡ Chi-square test.

To investigate the differential effect of acupoint application based on baseline pain severity, we compared the magnitude of change (*Δ* = pre-treatment score − post-treatment score) among the three subgroups using a one-way ANOVA. The analysis revealed that the improvement varied significantly depending on the initial pain level for all outcomes: daytime cough score (F(2,47) = 4.81, *p* = 0.013), nighttime cough score (F(2,47) = 6.74, *p* = 0.003), QoL score (F(2,47) = 5.12, *p* = 0.010), and VAS score (F(2,47) = 5.89, *p* = 0.005).

Post-hoc Tukey tests were conducted for pairwise comparisons. The improvement in daytime cough score was significantly greater in the severe pain subgroup (*Δ* = 2.92 ± 0.81) compared to the mild pain subgroup (*Δ* = 1.39 ± 0.83; *p* = 0.009). Similarly, the severe pain subgroup showed a significantly larger reduction in nighttime cough score (Δ = 3.10 ± 1.04) than the mild pain subgroup (Δ = 2.04 ± 0.58; *p* = 0.003). For QoL, the increase was more pronounced in the severe pain subgroup (Δ = 7.80 ± 3.85) than in the mild pain subgroup (Δ = 5.33 ± 2.86; *p* = 0.024). Concurrently, the reduction in VAS score was also greater in the severe pain subgroup (Δ = 3.39 ± 2.58) compared to the mild pain subgroup (Δ = 1.78 ± 2.15; *p* = 0.017).

No significant differences in the magnitude of improvement were observed between the moderate and severe pain subgroups or between the moderate and mild pain subgroups for any of the outcome measures (all *p* > 0.05). Furthermore, the overall response rate did not differ significantly among the three pain severity subgroups (85% vs. 82% vs. 84%, χ^2^ = 0.08, *p* = 0.961).

### Multivariate analysis of treatment efficacy

The results of the multivariate analyses, adjusting for baseline outcome scores and history of allergy, are summarized in Table 4. After adjustment, the acupoint application therapy (observation group) remained a statistically significant independent factor associated with superior outcomes across all measures.

**Table 4:** Multivariate analysis of the independent effect of acupoint application therapy on primary outcomes.

Outcome measure	Model type	Adjusted mean difference (aMD) or adjusted odds ratio (aOR)*	95% confidence interval	*p* value
Post-treatment daytime cough score	Multiple linear regression	aMD = −0.21	−0.30 to −0.12	<0.001
Post-treatment nighttime cough score	Multiple linear regression	aMD = −0.88	−1.06 to −0.70	<0.001
Post-treatment QoL score	Multiple linear regression	aMD = 1.21	0.10 to 2.32	0.033
Post-treatment VAS score	Multiple linear regression	aMD = −1.58	−2.14 to −1.02	<0.001
Treatment response	Multiple logistic regression	aOR = 4.72	1.52 to 14.67	0.007

All models are adjusted for the baseline value of the respective outcome and history of allergy. aMD: adjusted mean difference; aOR: adjusted odds ratio; QoL: quality of life; VAS: visual analogue scale. *For continuous outcomes (cough scores, QoL, VAS), the statistic is aMD (observation group vs. control group). For the binary outcome (response rate), the statistic is aOR (observation group vs. control group).

Specifically, compared to the control group, the observation group was associated with significantly lower post-treatment daytime cough scores (aMD = −0.21, 95% CI: −0.30 to −0.12; *p* < 0.001) and nighttime cough scores (aMD = −0.88, 95% CI: −1.06 to −0.70; *p* < 0.001). Similarly, the observation group was independently associated with higher QoL scores (aMD = 1.21, 95% CI: 0.10 to 2.32; *p* = 0.033) and lower VAS scores (aMD = −1.58, 95% CI: −2.14 to −1.02; *p* < 0.001).

Furthermore, in the multiple logistic regression model, patients in the observation group had significantly higher odds of achieving a treatment response after adjusting for covariates (aOR = 4.72, 95% CI: 1.52 to 14.67; *p* = 0.007).

### Response rate and adverse reactions

The overall response rate in the observation group was 85%, higher than the 81% in the control group (*p* < 0.05). At the same time, there were no differences in response rates among patients with different pain severity in the observation group. Moreover, none of the participants in this study experienced adverse reactions.

## Discussion

In this study, both the observation and control groups showed a decrease in daytime and night-time scores, an increase in QoL scores and a decrease in VAS scores compared with pre-treatment scores. Overall, the acupoint application group reported superior efficacy. Meanwhile, within the observation group, the efficacy of acupoint application in patients with severe pain was greater than that in those with mild pain, with more substantial improvements in cough symptoms, QoL and pain. This suggests that patients with severe pain may respond better to acupoint application therapy. This further indicates that the mechanism of acupoint application is multifaceted. Inflammation is an important cause of pain. Patients with severe pain often have more severe inflammation. Acupoint Application therapy can decrease peripheral blood eosinophilic granulocyte count (19, 20) and stimulate the body’s immunity and reduce allergic states (21). This may be the reason why acupoint application has better therapeutic effects in patients with severe pain.

Acupuncture can substantially reduce the frequency of coughing and minimise damage to lung tissue, a mechanism that may be related to the inhibition of inflammation (22). Acupoint application therapy has demonstrated excellent effects on cough caused by chronic obstructive pulmonary disease and chronic bronchitis, improving not only cough symptoms but also lung function (23–25). Chinese herbal medicine can regulate the nervous and endocrine systems and enhance immune function, thereby improving the activity of various tissues and organs to help restore normal physiological functions (25).

Commonly used herbs for acupoint application include white mustard seed, *Corydalis yanhusuo*, *Euphorbia kansui* and asarum. Specifically, white mustard seed is beneficial for warming the lungs and reducing phlegm and is used for cold phlegm, asthma, cough and fluid retention; *Corydalis yanhusuo* is effective in activating blood circulation, promoting qi and relieving pain, while also guiding other herbs into the meridians; *Euphorbia kansui* has properties of removing water retention by purgation, dispersing swelling and dissipating binds, supporting the movement of water and dampness through the meridians and promoting phlegm clearance; asarum can expel wind, disperse cold and warm the lungs to reduce watery phlegm and is effective for stagnant fluid retention, reversed flow of qi, cough and wheezing caused by cold phlegm (26).

In this study, the improvement in cough symptoms in the observation group was more substantial than in the control group, demonstrating the good efficacy of acupoint application and aligning with previous studies (27). The improvement of cough symptoms by acupoint application may be related to the regulation of inflammatory mediators (28). Research has shown that acupoint application therapy can improve lung function, suggesting that the relief of cough symptoms may be mediated through enhanced lung performance (29). Furthermore, acupoint application has been shown to improve patients’ QoL (30). In this study, the improvement in cough symptoms and pain resulting from acupoint application appears to be a key contributor to the observed enhancement in QoL. Moreover, acupoint application has demonstrated satisfactory efficacy for various types of pain (31, 32), with this study further validating its effectiveness in pain relief. In this regard, the realisation of therapeutic efficacy depends on the appropriate selection of acupoints and the pharmacological properties of the herbs used. Crucially, the multivariate regression analyses confirmed that the acupoint application therapy was an independent and significant factor associated with these improved outcomes, even after adjusting for baseline scores and the imbalance in allergy history.

The acupoints selected in this study included Danzhong-CV17, Tiantu-CV22, Shenque-CV8, Qihai-CV6, Guanyuan-CV4 and Zhongwan-CV12, which are primarily used for the lung meridian and related respiratory diseases (33–35). The combination of multiple acupoints can warm the yang-qi and strengthen the spleen, improving the functions of the spleen and stomach (36), making it suitable for cough due to qi deficiency of the lung and spleen.

In terms of herb selection, *Astragalus membranaceus* and *Codonopsis pilosula* can tonify the lung and spleen, alleviating symptoms such as shortness of breath, wheezing and spontaneous sweating caused by the syndrome of qi deficiency (37). The combination of *Carthamus tinctorius* and *chuanxiong* is effective in activating blood circulation and relieving pain (38). In this study, the improvement in symptoms and QoL among patients with severe pain was more pronounced compared with those with mild pain, possibly due to the greater potential for improvement in patients with severe symptoms or their better response to transdermal absorption of the medication. These differences will be explored in greater depth in future research.

Notably, this study has some limitations: (1) data on recurrence rates were unavailable due to the absence of long-term follow-up, making it impossible to assess the long-term efficacy of acupoint application therapy and (2) all patients included in this study were from the same hospital, which compromises the representativeness of the findings; and (3) although multivariate analyses were performed to control for key confounders, the possibility of residual confounding from unmeasured variables cannot be entirely ruled out.

## Conclusion

Acupoint application therapy demonstrates satisfactory clinical efficacy in children with chronic cough, with more pronounced benefits observed in those experiencing severe pain.

## Data Availability

The original contributions presented in the study are included in the article/supplementary material, further inquiries can be directed to the corresponding author.

## Footnotes

1
https://www.isrctn.com/
